# Supplementary material for: Predictive Role of Serum Thyroglobulin after Surgery and before Radioactive Iodine Therapy in Patients with Thyroid Carcinoma
Source: Cancers (Basel). 2023 May 30;15(11):2976. doi: 10.3390/cancers15112976 (PMC10251887; doi:10.3390/cancers15112976)
Supplement: Supplementary file 1 [file cancers-15-02976-s001.zip › cancers-2349542-supplementary.pdf]

**Supplementary Figures**

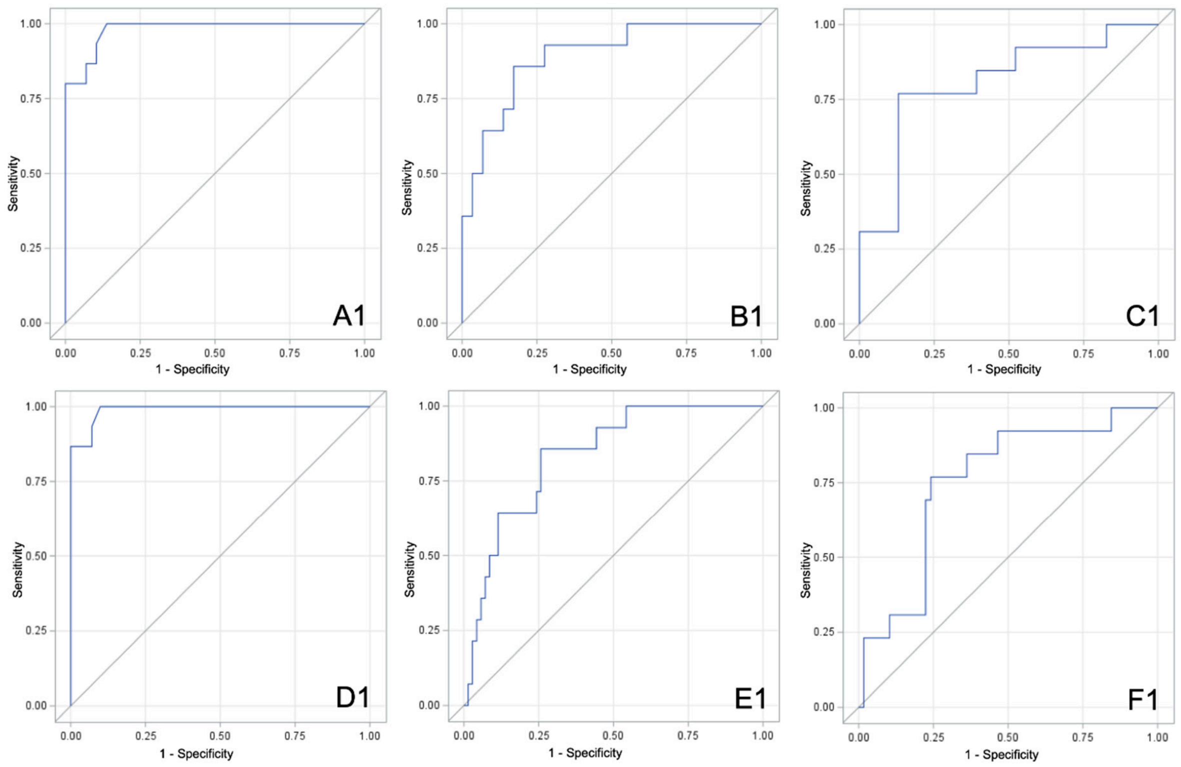


**Supplementary Figure S1:**

ROC curves of serum thyroglobulin at 30 days before RAI ($Tg_{-30}$) (A1), on the day of RAI ($Tg_0$) (B1) and 7 days after RAI ($Tg_{+7}$) (C1) when comparing nodal disease (ND) vs. non-evidence of structural or biochemical disease + intermediate ATA risk (NED-I) or when comparing nodal disease (ND) vs. non-evidence of structural or biochemical disease + low ATA risk (NED-L), (D1, E1, F1). A1 - AUC (95%CI): 98.1 (95.1 to 100); B1 - AUC (95%CI): 89.2 (79.2 to 99.2); C1 - AUC (95%CI): 80.6 (64.7 to 96.5); D1 - AUC (95%CI): 99.0 (97.4 to 100); E1 - AUC (95%CI): 83.6 (73.3 to 93.8); F1 - AUC (95%CI): 75.5 (61.3 to 89.6).

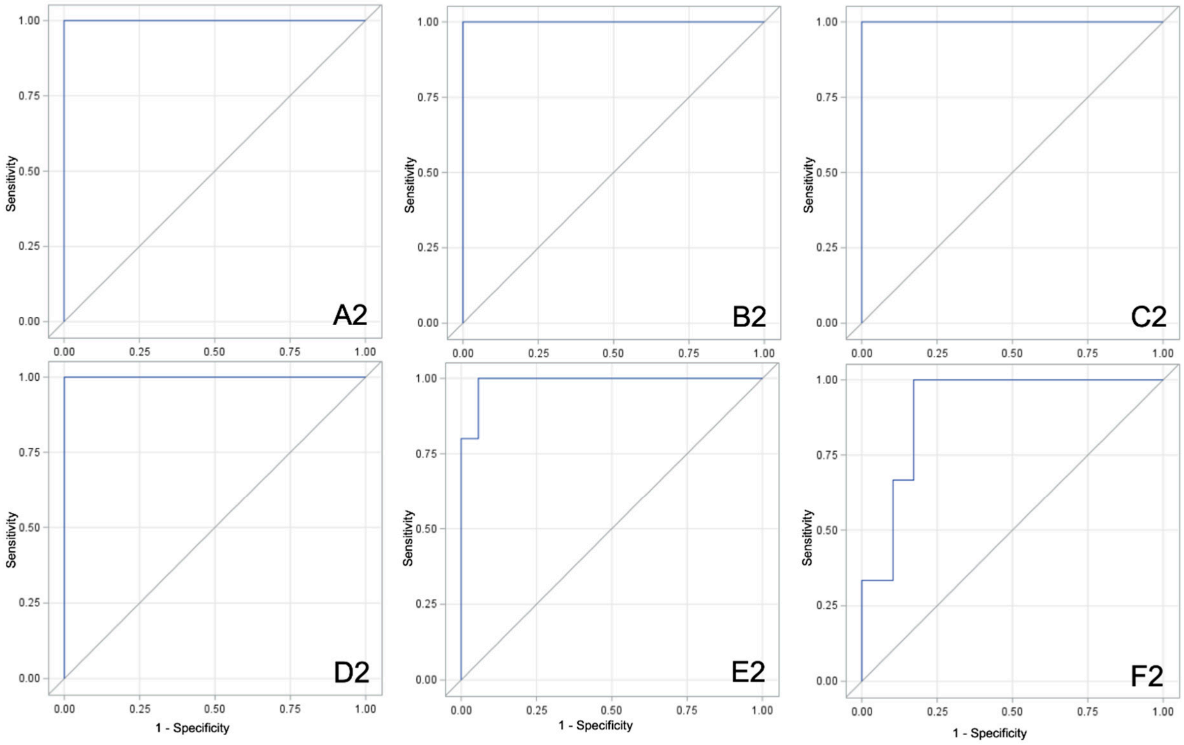


**Supplementary Figure S2:**

ROC curves of serum thyroglobulin at 30 days before RAI ($Tg_{-30}$) (A2), on the day of RAI ($Tg_0$) (B2) and 7 days after RAI ($Tg_{+7}$) (C2) when comparing distant disease (DD) vs. no evidence of structural or biochemical disease + intermediate ATA risk (NED-I) or when comparing distant disease (DD) vs. no evidence of structural or biochemical disease + low ATA risk (NED-L), (D2, E2, F2). A2 - AUC (95%CI): 100 (100 to 100); B2 - AUC (95%CI): 100 (100 to 100): C2 - AUC (95%CI): 100 (100 to 100): D2 - AUC (95%CI): 100 (100 to 100); E2 - AUC (95%CI): 99.2 (97.4 to 100); F2 - AUC (95%CI): 93.4 (85.3 to 100).
